# Protective effect of Gelofusine against cRGD-siRNA-induced nephrotoxicity in mice

**DOI:** 10.1080/0886022X.2018.1450761

**Published:** 2018-04-05

**Authors:** Wenjie Liao, Yixin Qin, Lumin Liao, Bohong Cen, Zhuomin Wu, Yuanyi Wei, Zhen Wang, Guoxian Li, Aimin Ji

**Affiliations:** Department of Pharmacy, Zhujiang Hospital, Southern Medical University, Guangzhou, China

**Keywords:** Gelofusine, CRGD-siRNA, nephrotoxicity, SiRNA delivery, tumor therapy, toxicology

## Abstract

Based on successful targeting to the αvβ3 integrin of cyclic arginine–glycine–aspartic acid (cRGD), cRGD-conjugated small interfering RNA (siRNA) exhibits tumor targeting and has become a new treatment strategy for solid tumors. However, the nephrotoxicity caused by its renal retention limits its clinical application. Here, we evaluated the protective effect of Gelofusine against cRGD-conjugated siRNA-induced nephrotoxicity in mice. Male Kunming mice (six per group) were either co-injected with Gelofusine and cRGD-siRNA or injected with cRGD-siRNA alone. After administration of these treatments five times, creatinine and blood urea nitrogen (BUN) levels were determined. Hematoxylin–eosin staining (HE staining) and transferase dUTP nick end labeling (TUNEL) analysis were used to compare the difference in renal damage between the groups. Additionally, fluorescence imaging was used to observe the distribution of cRGD-siRNA in vivo. The group co-injected with Gelofusine and cRGD-siRNA displayed lower creatinine and BUN levels than the cRGD-siRNA-alone group and showed less renal damage upon HE staining and TUNEL analysis. Gelofusine decreased the retention time and accelerated the elimination of cRGD-siRNA from the organs, as observed in the fluorescence images. These data indicate that Gelofusine significantly increased the excretion of cRGD-conjugated siRNA and reduced the associated renal damage.

## Introduction

Small interfering RNA (siRNA) is an important component of the RNA interference (RNAi) machinery, which leads to specific gene silencing by inhibiting specific mRNA translation. SiRNAs show promising therapeutic effects in a broad range of diseases from communicable diseases to cancers [1]. However, a series of obstacles must be overcome before the clinical application of siRNA, and the lack of targeting is a major issue. Several delivery systems have been successfully explored for safely carrying specific siRNAs to targeted sites, including receptor-specific ligands [2,3], receptor-specific agonists [4] and the assembly of siRNAs into nanoparticles with lipids [5] and cationic polymers [6].

Integrins are a type of cell adhesion molecule that establishes a connection between the nucleus and external environment. The integrin–αvβ3 receptor, which is overexpressed in the endothelial cells of the tumor neovasculature, plays an important role in angiogenesis and tumor metastasis. Researchers have found that cyclic arginine–glycine–aspartic acid (cRGD) peptides can specifically bind to integrin–αvβ3 receptors expressed in the endothelial cells of the tumor neovasculature [7]. SiRNAs targeting vascular endothelial growth factor receptor (VEGFR) mRNA can inhibit tumor angiogenesis. Hence, we conjugated the siRNA to cRGD, which was proven to exhibit tumor targeting and to inhibit tumor growth effectively [8].

However, cRGD-conjugated siRNA shows glomerulus and renal tubule toxicity after long-term administration, which limits its clinical application. Following glomerular filtration, oligopeptides are reabsorbed by tubular proximal cells and then proteolytic digestion occurs in lysosomes. It is known that the reabsorption of low-molecular weight proteins and peptides is mediated by the megalin/cubilin system [9]. Both megalin and cubilin are members of the low-density lipoprotein (LDL) receptor family and play an important role in stabilizing calcium levels in organisms as well as the reabsorption of peptides, vitamin D-binding protein and some types of hormones [10,11]. The reabsorption of cRGD depends upon megalin/cubilin system.

When siRNA that conjugated with oligopeptides are reabsorbed by megalin/cubilin system, the nephrotoxicity may be caused by Toll-like receptors (TLRs) which are expressed in kidney tubules. TLRs are a receptor family that recognize pathogen-associated molecular patterns (PAMPs) and promote the activation of immune leucocyte [12]. Researches have shown that TLR3 has high expression on renal tubule epithelial cells [13,14]. It is noteworthy that the activation of TLR3 is modulated by single- and double-stranded RNA sequences [15,16]. The activated TLR3 lead to the induction of antiviral and proinflammatory cytokines and dendritic cell maturation, thus eliciting innate immune responses [15,17]. The reason why cRGD-conjugated siRNA lead to nephrotoxicity may be the siRNA trigger immune responses through TLRs after tubular reabsorption.

It has been reported that injection of Gelofusine, which was usually used to increase blood volume, effectively increases the excretion of megalin ligands through promoting urine elimination of the ligands [18].

In this study, we discussed the protective effect of Gelofusine on cRGD-conjugated siRNA nephrotoxicity in mice through blood tests, pathological examination, transferase dUTP nick end labeling (TUNEL) staining and fluorescence imaging. The study was performed in 6- to 8-week-old male Kunming mice.

## Materials and methods

### Preparation of cRGD-conjugated siRNA

The siRNA sequence employed for our experiments was human VEGFR2 siRNA. The sense strand was 5′-mGmAmGAACCUCACAUGGUmAmCmA dTdT-3′ and the antisense strand was 5′-mUmGmUACCAUGUGAGGU-UmCmUmC dTdT-3′. Cy5 phosphoramidites (RiboBio Co., Ltd., Guangzhou, China) were attached to the 5′-ends of the siRNA antisense strands. The synthesis and purification of the sense strand and the antisense strand were based on standard oligonucleotide synthesis and deprotection protocols [19]. Cyclo (Arg-Gly-Asp-D-Phe-Lys[PEG-MAL]) (cRGD) peptides, where PEG is 8-amino-3,6-dioxaoctanoic acid and MAL is β-maleimidopropionic acid, were synthesized by Peptide International (Louisville, KY). The conjugation of cRGD-siRNA was performed as follows. The 5′-thiol-modified siRNA sense strand was dissolved in HEPES-KOH buffer (pH 7.2) and cRGD was then added. N_2_ was used to saturate the solution to remove air and the Michael reaction was performed at 4 °C. The resulting solution was filtered via centrifugation (MW = 3000) and then washed three times with RNase-free water to remove excess cRGD.

Lyophilization was performed to produce cRGD-single-stranded (ssRNA) and the final products were detected via liquid chromatography–mass spectrometry (LC-MS) and high-performance liquid chromatography (HPLC). Subsequently, the cRGD-ssRNAs were mixed with antisense RNAs in annealing buffer (Tris-HCl pH 7.4, NaCl and ethylenediaminetetraacetic acid), then thermally denatured at 95 °C for 3 min and cooled down to room temperature. Ultrafiltration was performed for desalting and the solution was freeze-dried.

### Animal handling and serum chemistry analysis

Six- to eight-week-old male Kunming mice (20–25 g) were obtained from the Experimental Animal Center of Sun YatSen University (Guangzhou, China) and raised with free access to rodent food and water. All animal experiments were carried out in accordance with the Laboratory animal guidelines of welfare ethical review, which was approved by the Laboratory Animal Welfare ad Ethics Committee.

Eighteen mice were distributed in three groups (*n* = 6/group). Each animal in group 1 received a tail vein injection of cRGD-siRNA (5 nmol) and each animal in group 2 was co-injected with cRGD-siRNA (5 nmol) and Gelofusine (4 mg), while the mice in group 3 (the control group) were injected with saline (100 µl). All the mice were dosed continuously five times at intervals of 48 h. On the second day after the last injection, all mice were weighed and euthanized, and their blood and kidneys were collected. The serum was then separated, and creatinine and blood urea nitrogen (BUN) levels were determined with an automatic blood instrument.

### Renal index and histological examination

The kidneys were weighed and the renal index (RI) was calculated using the formula: RI = weight of both kidneys (g)/animal weight (g) × 100%. The kidneys were fixed in 4% paraformaldehyde for 8–12 h, and the tissues were then cut into 4-μm sections. The sections were stained with HE and observed using a photo-electric microscope.

### TUNEL assay

The terminal deoxynucleotidyl TUNEL assay was used to detect cell apoptosis. Photographs were taken and cell numbers were counted. Ten views were chosen for each section and the apoptotic index (AI) was calculated using the following formula: AI = number of apoptosis-positive cells/number of total cells ×100%.

### Distribution of cRGD-siRNA in vivo

Six- to eight-week-old male Kunming mice (20–25 g) were co-injected intravenously with cRGD-siRNA-Cy5 (1 nmol/20 g) and Gelofusine (4 mg) or were injected intravenously with Cy-5-labeled cRGD-siRNA (1 nmol/20 g) alone at single doses (*n* = 12). Half the mice in each group were euthanized 24 h after treatment and half the mice were euthanized 48 h after treatment. The major organs were excised and imaged using an IVIS spectrum imaging system immediately (Cy5: λ ex = 640 nm, λ em = 680 nm).

### Statistical analysis

Statistical analysis was carried out using one-way analysis of variance (ANOVA) (SPSS 19.0 software, Armonk, NY, USA). Data are expressed as the mean ± SD and *p* values of .05 or less were accepted as statistically significant data. The unpaired *t*-test was employed to compare the two groups.

## Results

### Synthesis and identification of cRGD-siRNA

**Figure 1.**
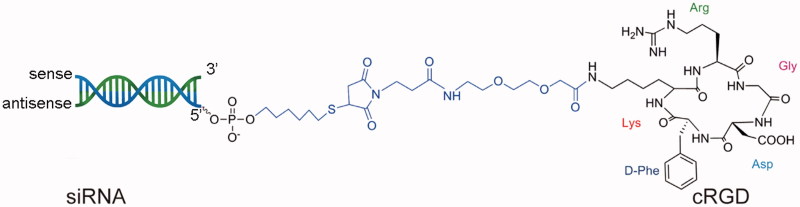
Diagram of cRGD-siRNA. The cRGD (right) is covalently conjugated to the 5′-end of the siRNA sense strand (left) through a thiol-maleimide linker.

**Figure 2.**
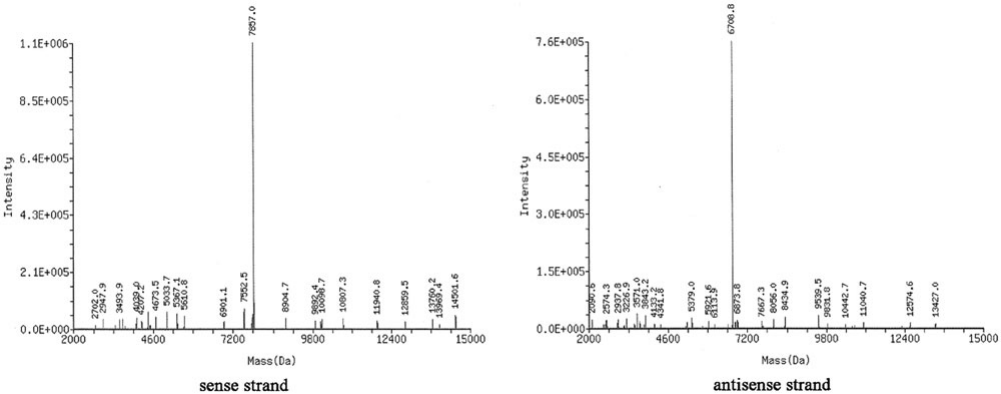
Identification of cRGD-siRNA via LC-MS. The molecular mass of cRGD-conjugated sense strand siRNA was 7857.0 Da, which was close to the theoretical mass of 7856.4 Da. The measured mass of the antisense strand was 6708.8, which was also close to the theoretical mass of 6708.2.

The cRGD peptide was conjugated to the 5′-phosphate of the siRNA sense strand with a thiol-maleimide linker. A schematic diagram of the cRGD-siRNA conjugates is presented in Figure 1. LC-MS results revealed that the molecular mass of the cRGD-conjugated siRNA sense strand was 7857.0 Da, which was close to the theoretical mass of 7856.4 Da, as shown in Figure 2. The results indicated that the synthetic products were consistent with the theoretical molecules. The purity of cRGD-conjugated siRNA reached 81.3% according to the HPLC results.

### Serum Cr and BUN levels

**Figure 3.**
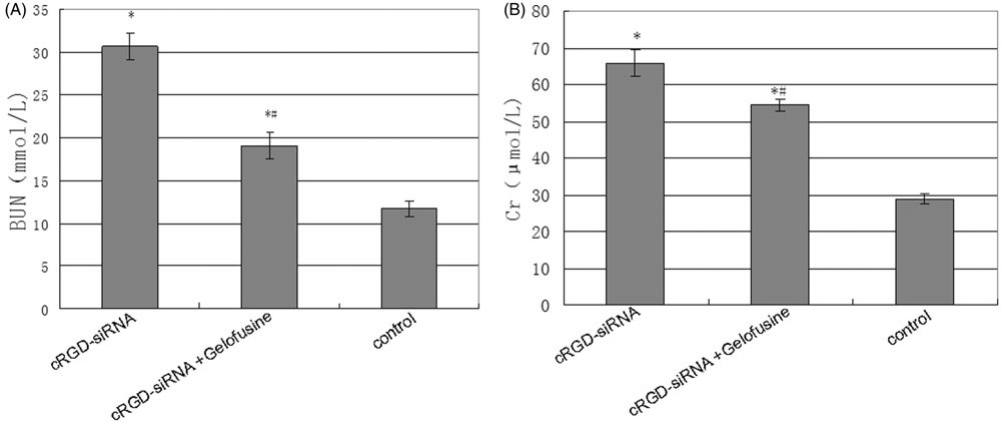
Levels of serum urea nitrogen and creatinine in the different groups. (A) Serum urea nitrogen. (B) Serum creatinine. On the second day after the last injection, blood samples were collected and analyzed with an automatic blood instrument. Data are expressed as the mean ± SD. *p < .05 versus the control group and #*p <* .05 versus the cRGD-siRNA group.

The injection of cRGD-siRNA significantly increased serum creatinine (serum Cr) and BUN levels compared with those in the saline control group. Co-injection of Gelofusine strongly decreased the cRGD-conjugated siRNA-induced increases in serum Cr and BUN compared with those in the cRGD-siRNA group (Figure 3).

### Histopathology and TUNEL assay

**Figure 4.**
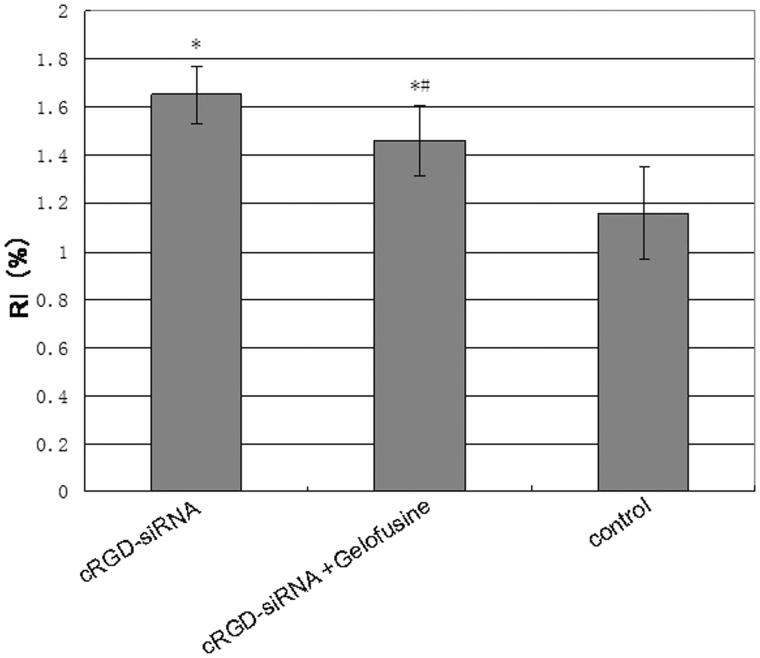
Comparison of the renal index (RI) between different groups. After 48 h of the last treatment, we weighed and euthanized the mice. We then separated and weighed their kidneys and calculated the RI. Data are expressed as the mean ± SD. *p < .05 versus the control group and #*p <* .05 versus the cRGD-siRNA group.

Compared with the group co-injected with Gelofusine, the RI of the cRGD-siRNA group was significantly increased (*p* < .05), which indicated that the mice in the cRGD-siRNA group exhibited weight loss and hydronephrosis, as shown in Figure 4.

**Figure 5.**
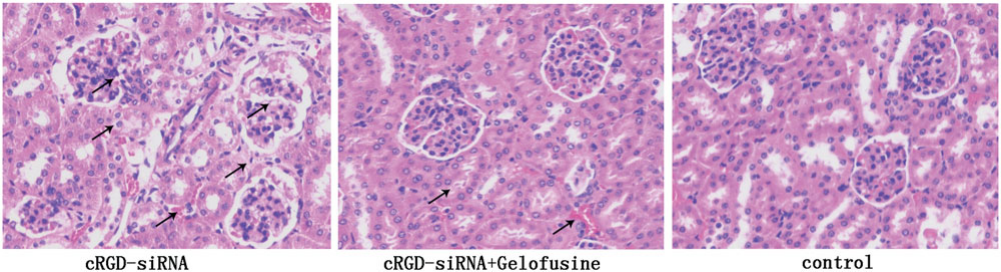
HE staining of the kidney tissues of the mice. The kidneys were fixed in 4% paraformaldehyde and then cut into 4-μm sections. The sections were stained with hematoxylin and eosin and observed using an electric microscope (×400).

The hematoxylin–eosin staining (HE) staining results showed that the cRGD-siRNA group exhibited edema and coagulation necrosis in many tubules as well as glomerular atrophy and cystic dilatation. In contrast, the renal damage of the group co-injected with Gelofusine was much less severe, as shown in Figure 5. The results indicated that Gelofusine can effectively reduce the renal damage caused by cRGD-siRNA.

**Figure 6.**
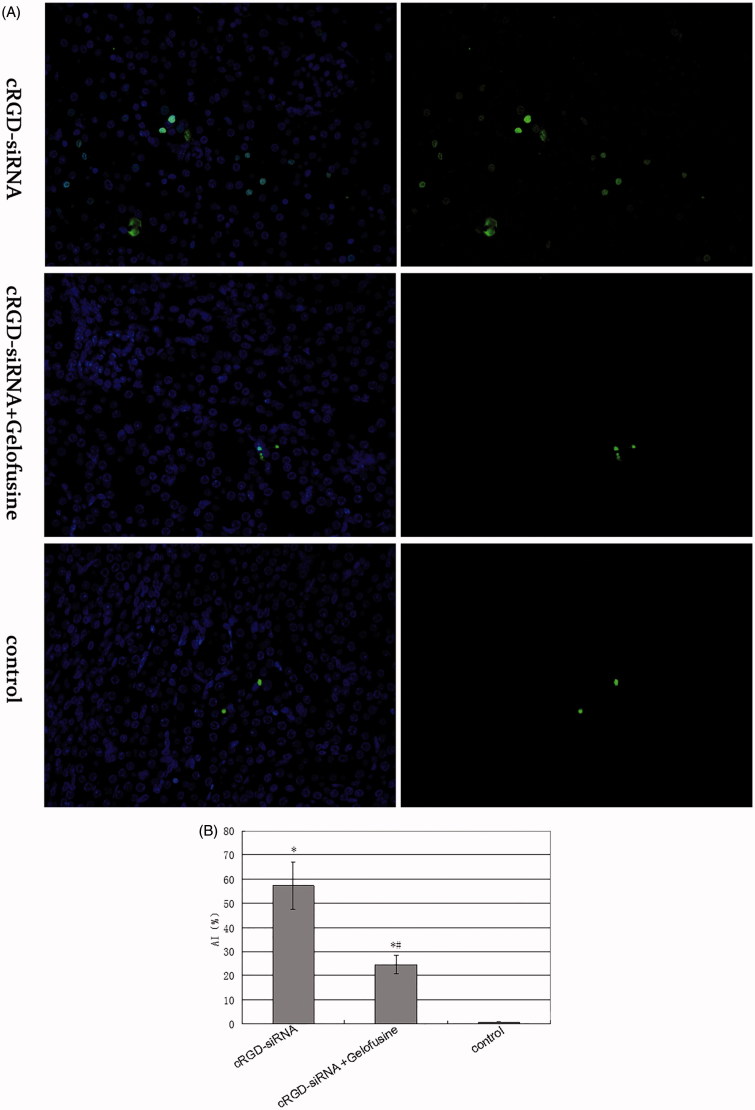
Apoptosis was detected in the kidney via TUNEL staining. (A) All the cells were displayed under 460 nm fluorescence, and only the apoptotic cells were displayed under 520 nm fluorescence (×400). (B) Apoptotic indexes (AI) of different groups. Ten views were chosen for each section and AI was calculated using the following formula: AI = number of apoptosis-positive cells/number of total cells × 100%. Data are expressed as the mean ± SD. *p < .05 versus the control group and #*p <* .05 versus the cRGD-siRNA group.

The results of the TUNEL assay revealed a greater number of apoptotic cells in the cRGD-siRNA group compared with those in the Gelofusine co-injection group, with the corresponding AI reaching 57.3 ± 9.8 (Figure 6(A,B)).

### Distribution of cRGD-siRNA in vivo

**Figure 7.**
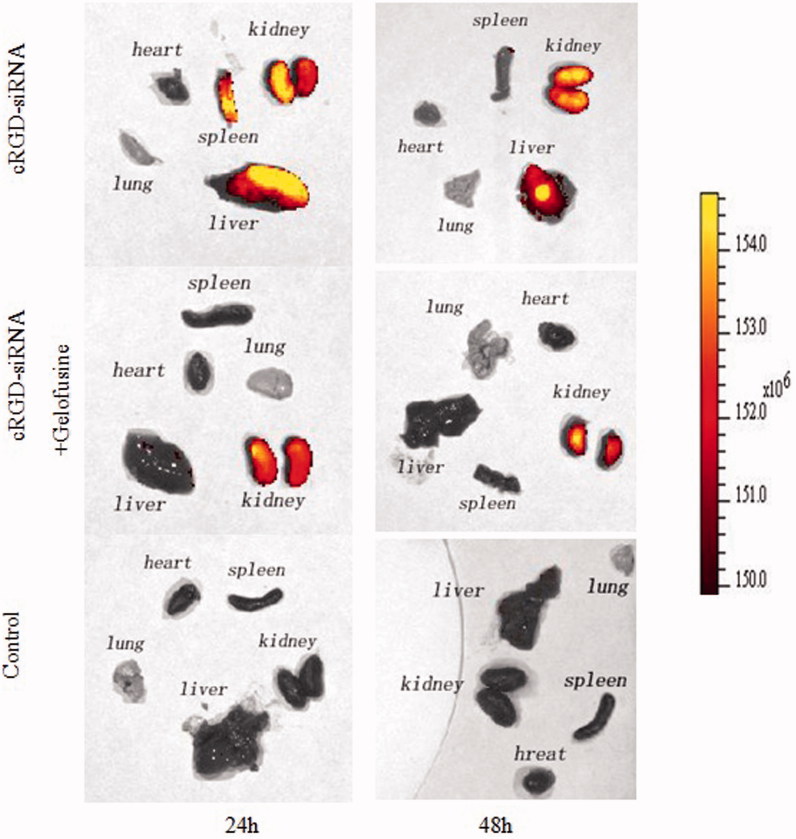
Bio-distributions of cRGD-siRNA in vivo. Mice were intravenously co-injected with Cy-5 labeled cRGD-siRNA (1 nmol/20 g) and Gelofusine (4 mg) or were injected with cRGD-siRNA-Cy5 alone at single doses. The mice were euthanized at 24 h or 48 h after administration, and their major organs were excised and imaged using an IVIS imaging system (Cy5: λ ex = 640 nm, λ em = 680 nm). All images were scaled to the same maximum and minimum color values.

At 24 and 48 h after injection, abundant Cy5 fluorescence was observed in the kidneys and livers of the mice in the cRGD-siRNA group, and some fluorescence was also present in the spleen. In contrast, upon co-injection with Gelofusine, Cy5 fluorescence was much weaker in the kidneys, and there was little fluorescence in the liver. The results showed that Gelofusine expedited the elimination of cRGD-siRNA and alleviated the renal retention of cRGD-siRNA (Figure 7).

## Discussion

The integrin–αvβ3 receptor plays an important role in angiogenesis and tumor metastasis and is overexpressed in the endothelial cells of the tumor neovasculature. RGD was developed to target tumors that express integrin αvβ3 as a drug delivery system and a contrast agent for nuclear medicine and optical imaging [20,21]. VEGFR2 siRNA which conjugated with cRGD targets the tumor neovasculature and then plays a role in inhibiting angiogenesis. Nevertheless, the associated toxicity to vital tissues, such as the kidneys, limits the dose and further clinical application of cRGD-siRNA. In the present study, we found that the use of Gelofusine significantly reduced the renal damage induced by cRGD-conjugated siRNA, which was consistently reflected in the serum chemistry analysis and the results of histopathology, TUNEL assays as well as fluorescence imaging. Oligopeptides are easily filtered in glomeruli and then reabsorbed in proximal tubules. The reabsorption of conjugated siRNA results in the activation of TLRs in the renal cortex and, thus, renal toxicity. Reducing reabsorption and increasing excretion are crucial for reducing the nephrotoxicity of peptide drugs.

Many homologs of toll, termed Toll-like receptors, were demonstrated to distinguish PAMPs and to trigger innate immune responses [17]. All TLRs activate NF-kB through the TRIF- or MyD88-dependent pathway or both [22,23]. The activation of NF-kB inflammatory pathway is significant for inducing innate immune responses, such as the induction of inflammatory cytokines, cell proliferation and apoptosis [23]. Double-stranded siRNA is the ligand of TLR3 which has a relatively high expression on renal tubule epithelial cells [13–16]. CRGD-conjugated siRNA activates TLR3 after tubular reabsorption. As the HE results show, cRGD-siRNA results in renal damage such as edema and falling of renal tubular epithelial cells, glomerular mesangial proliferation and erythrocyte effusion.

Megalin and cubilin, which exhibit higher expression levels in the proximal tubules and relatively lower expression levels in the glomeruli, are endocytic membrane receptors that have been described as crucial in the tubular reabsorption of peptides [10]. Cubilin combines with megalin via cationic sites and takes part in endocytosis only in the presence of megalin [24]. Megalin interacts with intracellular proteins, facilitated by adaptors, and thereby regulates cell pinocytosis and signal transduction. Some ligands bind to megalin via cationic sites [25]; thus, cationic compounds such as lysine or other positively charged amino acids can serve as competitive inhibitors [26]. As a result, the reabsorption of megalin ligands is effectively reduced. In fact, co-injection of lysine and arginine has become a necessary procedure when using 90Y-labeled or 177Lu-labeled somatostatin analogs [27]. However, a large infusion of cationic amino acids might cause hyperkalemia or cardiac toxicity [28]. In contrast, the carbohydrate-based plasma expander hydroxyethyl starch (HES) failed to reduce the kidney retention of radiolabeled octreotide [29]. Other plasma expanders, such as the lipid solutions Medialipid and Voluven, have also been found to exert no significant effect on the reduction of renal retention [30].

Gelofusine, also known as succinylated gelatin injection, consists of succinylated bovine gelatin molecules. It has been proven that Gelofusine is a safe plasma expender and few anaphylactic reactions have been reported [31]. Researchers found that Gelofusine could decrease the renal retention of megalin ligands [18,32]. The renal uptake of octreotide can be reduced by 46% using Gelofusine [33], and surprisingly, the tumor uptake of radiopeptides is not affected [34]. Gelofusine, which is rich in lysine residues and praline, may offer abundant cationic amino acids after injection and helps competitively saturate the megalin system; therefore, the co-injection of Gelofusine reduces the reabsorption of cRGD-siRNA.

In the present study, all the results showed significant differences between the saline group and the group co-injected with cRGD-siRNA and Gelofusine, indicating that although Gelofusine reduced the renal toxicity of cRGD-conjugated siRNA, Gelofusine could not completely prevent renal toxicity. A possible explanation might be that Gelofusine did not completely block the megalin/cubilin system, which is inadvisable. In addition to mediating the nephrotoxicity of some proteins, megalin plays an important role in signal transduction. Megalin knockout mice might face barriers to the reabsorption of protein and exhibit a lack of serum vitamin D and hypocalcemia [35]. Co-infusion of Gelofusine and lysine further improves the reduction of the renal retention of radiopeptides [34]. The combination of Gelofusine and other renal-protective compounds might completely prevent the renal toxicity induced by cRGD-siRNA, but further research is required for validation of this hypothesis.

In conclusion, our findings show that the plasma expander Gelofusine exerts a protective effect against the renal toxicity caused by cRGD-conjugated siRNA. This study may provide support for preclinical and clinical studies involving cRGD-conjugated siRNA and broaden the clinical application of Gelofusine.
